# A New Exopolysaccharide from a Wood-Decaying Fungus *Spongipellis borealis* for a Wide Range of Biotechnological Applications

**DOI:** 10.3390/molecules28166120

**Published:** 2023-08-18

**Authors:** Michał Fornal, Monika Osińska-Jaroszuk, Magdalena Jaszek, Dawid Stefaniuk, Adrian Wiater, Iwona Komaniecka, Łukasz Matuszewski, Anna Matuszewska

**Affiliations:** 1 Department of Biochemistry and Biotechnology, Medical University of Lublin, 20-059 Lublin, Poland; 62662@365.uml.edu.pl; 2 Department of Biochemistry and Biotechnology, Institute of Biological Sciences, Maria Curie-Sklodowska University, 20-031 Lublin, Poland; monika.osinska-jaroszuk@mail.umcs.pl (M.O.-J.); magdalena.jaszek@mail.umcs.pl (M.J.); dawid.stefaniuk@mail.umcs.pl (D.S.); 3 Department of Industrial and Environmental Microbiology, Institute of Biological Sciences, Maria Curie-Sklodowska University, 20-031 Lublin, Poland; adrian.wiater@mail.umcs.pl; 4 Department of Genetics and Microbiology, Institute of Biological Sciences, Maria Curie-Sklodowska University, 20-031 Lublin, Poland; iwona.komaniecka@mail.umcs.pl; 5 Pediatric Orthopedic and Rehabilitation Clinic, Medical University of Lublin, 20-059 Lublin, Poland; lukasz.matuszewski@umlub.pl

**Keywords:** fungi, *Spongipellis borealis*, exopolysaccharide, proteolytic enzymes, laccase

## Abstract

Fungi are a unique natural resource rich in polysaccharides, proteins, and other components. Polysaccharides are considered one of the most important bioactive components in fungi. Increasing numbers of studies have confirmed that fungal polysaccharides have various biological activities. Given these facts, the main aim of this investigation was to carry out isolation, identification, and structural characterisation of a new polysaccharide (EPS) derived from laboratory-cultured vegetative mycelium of a new *Spongipellis borealis* strain isolated from the environment. The examination of monosaccharides in the EPS demonstrated that the isolated biopolymer was composed mainly of glucose, galactose, and mannose monomers. The analysis of the methylation of the studied polymer indicated that it contained mainly terminal, →3)-linked, →4)-linked, and →2,4)-linked hexoses. The effect of fungal polysaccharides on *S. borealis* proteolytic enzymes (pepsin, trypsin, and pycnoporopepsin) and laccase activity was determined for the first time. Incubation of the enzyme preparation and EPS showed an influence of EPS on the stability of these enzymes, compared to the control values (without EPS).

## 1. Introduction

*Spongipellis borealis*, also known as *Climacocystis borealis* (Fr.) Kotl. and Pouzar, is a large-fruited mushroom species belonging to the *Basidiomycota*, broadly widespread in boreal and boreal-mountainous forests [1]. This saprotrophic fungus (rarely parasitic) decays the wood of conifers and causes white rot of wood. It belongs to the family *Polyporaceae*, whose representatives are able to degrade both polysaccharide and lignin compounds in wood [2,3]. These mushrooms are also known to be a potential source of many bioactive compounds, such as triterpenoids, sterols, proteins, peptides, alkaloids, fatty acids, and polysaccharides [4].

A particularly interesting group of bioactive compounds in fungi are polysaccharides. They are divided into two groups: rigid fibres insoluble in water (cellulose, chitin, and lignin) and fibres that are soluble and loosely integrated with the fungal cell wall, filling the spaces between cells (β-glucan, α-glucan, and chitosan) [5]. Their biological activity depends on the chemical structure, conformation, and molecular weight. These polysaccharides are represented mainly by glucans, but they can also have a galactan or mannan structure [6]. Glucans and glycoproteins are components of fungal cell walls. These compounds shape cells and create a barrier protecting against environmental stress. The outer layer of the cell wall consists of glycoproteins, whereas the inner layer underneath is formed by polysaccharides: (1→3)-β-D-glucans, (1→6)-β-D-glucans, (1→3)-α-D-glucans, and chitin [7]. Polysaccharides with a linear structure and without long side chains are particularly active, due to their easier solubility and digestibility. Most often, there are β-(1→3) bonds in the main chain, and the branches are formed by β-(1→6) bonds. Polysaccharides with an additional peptide fragment are also known [6].

Due to their bioactive properties, polysaccharides can be used in medicine and biotechnology. Some immunomodulating, antitumor, antioxidative activity, including reduction in genetic material damage, of polysaccharides have been reported [8]. The most active modulators of the immune response are (1→3)-, (1→4)-, (1→6)-β-D-glucans and (1→4)-, (1→6), (1→3)-α-D-glucans [9]. They also have antibacterial and antiviral properties [10].

A very interesting aspect of the application of polysaccharides obtained from microbial sources, such as bacterial or fungal cultures, is the ability to modulate the properties of biocatalytically important enzymes, e.g., laccase, cellobiose dehydrogenase, or lipases. The addition of appropriately selected doses of polysaccharides may probably affect both the enzyme molecule itself by stabilising its structure and the conditions of the biochemical process catalysed by a given biomolecule. An example of such interactions may be the stabilising effect of carbohydrate preparations isolated from symbiotic bacteria of the genus *Rhizobium* on the activity of fungal laccase. As shown by previous analyses, bacterial EPSs increased the redox properties and stability of *C. unicolor* laccase in comparison to control samples [11]. A similar effect was observed in the case of *Pycnoporus sanguineus* cellobiose dehydrogenase (CDH). The addition of bacterial hydrocarbon preparations clearly enhanced the stability of enzyme activity [12]. The modification of the reaction mixture evidently increased the stability and operation activity of the enzyme (22 and 31% higher in the comparison to the values in samples without EPSs) [13]. Additionally, enhancement of ester synthesis by *Rhizomucor variabilis* lipase in the presence of fungal polysaccharides has been described [14].

Given these facts, the main aim of these investigations was to carry out isolation, identification, and structural characterisation of a new polysaccharide derived from laboratory-cultured vegetative mycelium of a new *S. borealis* strain isolated from the environment. Additionally, the application properties of the new exopolysaccharides, including their stabilising effect on the laccase and protease enzymes, were studied.

## 2. Results and Discussion

### 2.1. Genetic Characteristics of the Spongipellis borealis Strain 

The *S. borealis* strain used in this work was obtained from the culture collection deposited at the Department of Biochemistry of Maria Curie-Skłodowska University, Poland (strain no. 381). For genetic identification [15,16,17,18], a stationary culture of *S. borealis* was used. One 704 bp product was obtained from PCR with ITS1-ITS4 primers and subjected to direct sequencing. The complete sequences of these products (704 bp) indicated over 99% identity to the *S. borealis* ITS sequences. The sequence was deposited in GenBank under accession number KC293970.

### 2.2. Structural Analysis of Polysaccharides

**Figure 1:**
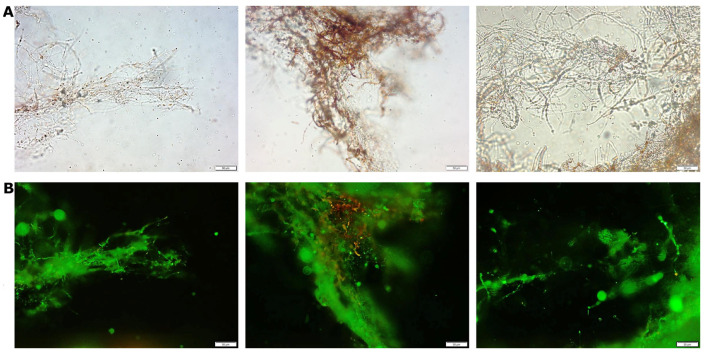
Detection of (1→3)-α-D-glucan in the hyphae of *S. borealis* by means of fluorophore-labelled antibodies (Clone IgM MOPC-104E). (**A**) Filaments in light microscopy, (**B**) fluorescent image of the same filaments. Twenty samples were observed, and typical images are presented. Scale bar = 50 µm.

Immunofluorescent labelling with specific antibodies was performed to detect (1→3)-α-D-glucan in *S. borealis* mycelium cell walls. The presence of (1→3)-α-D-glucan in the cell wall of *S. borealis* was imaged with the use of the fluorescent microscope (Figure 1). 

**Figure 2:**
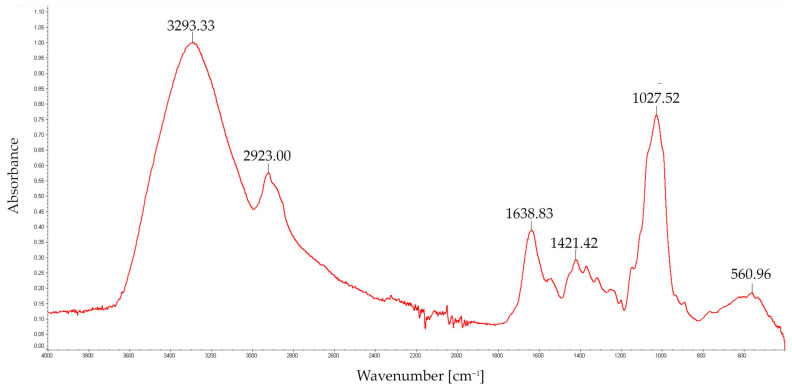
FT-IR spectrum of polysaccharides (EPS) obtained from *S. borealis*.

**Figure 3:**
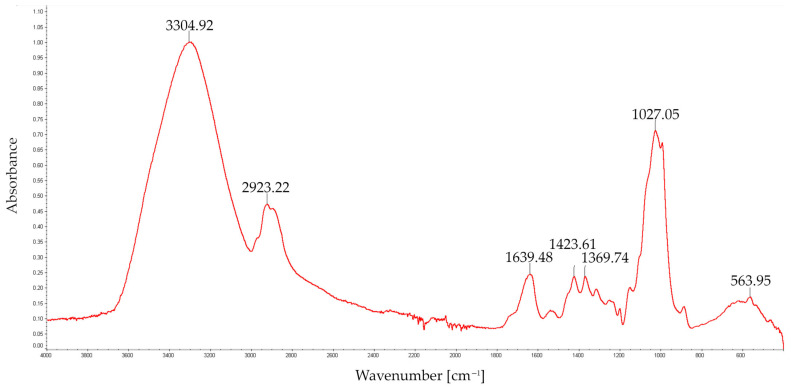
FT-IR spectrum of polysaccharides (EPS) obtained from *S. borealis*—after deproteination.

Structural analysis of the FT-IR spectra of EPS was carried out in the wavelength range of 4000 to 500 cm^−1^. As shown in Figure 2 and Figure 3, the FT-IR spectrum displayed the characteristic strong broad absorption in the range of 3000–3500 cm^−1^ corresponding to stretching vibration of O–H groups, characteristic of polysaccharide chains, and to asymmetric and symmetric stretching of the N–H bonds in amino groups [19]. An equally clear band between 1000 and 1100 cm^−1^ (i.e., 1027.52 cm^−1^ and 1027.05 cm^−1^), characteristic for the C–O stretching of –C–O–C– glycosidic linkages, indicates the presence of O-substituted glucose residues in the glucosidic chain [20]. Protein characteristic absorptions at 1638 cm^−1^ and 1421 cm^−1^ and at 3000–3500 cm^−1^ indicated the presence of a small amount of protein.

The band at 1638 cm^−1^ overlapped with the absorption of aromatics [21], probably polyphenols (C=C and C=O stretching vibrations), and most likely indicated the presence of polyphenols. Absorption bands between 1420 cm^−1^ and 1360 cm^−1^ were indicative of OH groups of phenolic compounds as well as a band at 1421 and 1423 cm^−1^ related to the aliphatic groups from phenolic pigments [22]. The FT-IR analysis of *S. borealis* EPS confirmed the spectral pattern typical of polysaccharides obtained from *Ganoderma applanatum*, *G. lucidum*, *Lentinus edodes*, and *Trametes versicolor* [23]. A similar profile of the spectrum was observed in a study of FT-IR spectra of glucans isolated from *Cerrena unicolor* cell walls [24].

The analysis of monosaccharides isolated from *S. borealis* demonstrated that hexoses glucose (Glc), galactose (Gal), and mannose (Man) were the main sugar components present in both samples (before and after the proteinase treatment) (Table 1). Small amounts of pentoses (ribose (Rib), arabinose (Ara), and xylose (Xyl)) and 6-deoxyhexose (identified as fucose (Fuc)) were also detected in both analysed samples. Additionally, a low amount of glucosamine was detected only in the crude sample (before the proteinase treatment). The amount of Glc was higher before the protein degradation and was estimated at over 78%. After the proteinase treatment, the amount of Man and Gal significantly increased compared to the crude sample. The content of galactose (Gal), glucose (Glc), and mannose (Man) in different molar ratios as the main polysaccharide components has also been confirmed by other authors [25,26,27]. In addition, other monosaccharides, e.g., arabinose (Ara), xylose (Xyl), fucose (Fuc), ribose (Rib), and rhamnose (Rha), have also been detected in fungal polysaccharides [28,29,30].

The GC–MS analysis of partly methylated alditol acetates revealed that the crude sample (1) contained mainly terminal, →3)-linked, →4)-linked, and →2,4)-linked hexoses (Table 2). In turn, sample 2 (after the proteinase treatment) contained almost two-fold higher amounts of →3)-linked and →2,4)-linked hexoses. In this sample, the amount of →4)-linked hexose II and →6)-linked hexose I dropped drastically, but the content of the terminal residues and branched →2,4,6)-linked hexoses was almost the same as in the crude sample.

### 2.3. Biochemical Analysis of Polysaccharides

The analysis of the chemical composition (concentration of proteins, total carbohydrates, reducing sugars, and total phenolic compounds) of the crude (sample 1) and deproteinated (sample 2) samples of S. borealis polysaccharides showed clear differences in the content of the analysed components (Table 3).

The crude EPS contained an evidently higher amount of total carbohydrates (673.85 µg/mL) than the deproteinated samples (363.88 µg/mL). The total amounts of polysaccharides in both samples were higher than those reported for *G. applanatum* (11.35 µg/mL), *Bjerkandera adusta* (42.54 µg/mL), *L. edodes* (50.81 µg/mL), or *Pleurotus ostreatus* (14.29 µg/mL) [31]. In addition, the concentration of phenolic compounds was significantly higher in the crude EPS (26.14 µg/mL) than in the deproteinated samples (9.89 µg/mL). The concentration of reducing carbohydrates after deproteinisation decreased significantly, suggesting that these are protein-bound sugars.

### 2.4. Effect of S. borealis EPS on Proteolytic Enzyme Activity

**Figure 4:**
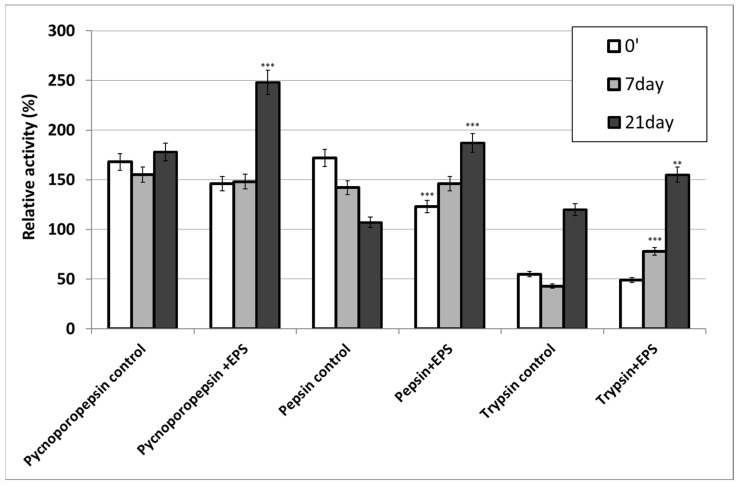
Proteolytic enzyme activity with or without EPS from *S. borealis*. The experiments were performed in triplicate. Average relative values (*n* = 3) and error bars are shown. Values with *p* ≤ 0.05 are significantly different (** *p* ≤ 0.01; *** *p* ≤ 0.001).

In the present study, the effect of *S. borealis* polysaccharides on the activity of proteolytic enzymes (pepsin, trypsin, and pycnoporopepsin) was determined for the first time. The proteolytic enzyme preparation was incubated in the presence or absence of EPS for 7 and 21 days at temperatures of 4 °C. The incubation of the enzyme preparation and EPS showed an influence of EPS on the stability of these enzymes compared to the control values (without EPS) (Figure 4). In samples incubated without exopolysaccharides, pepsin activity was significantly reduced (only 64.3% of basal activity was retained) after the 21-day storage of the enzyme at 4 °C. In turn, in the presence of EPS, the activity of pepsin increased by more than 50% after 21 days of incubation. An increase in the activity of pycnoporopepsin and trypsin was recorded after both 7 and 21 days of enzyme incubation with EPS, and the activities were higher than those obtained for the control variants. Similar results were obtained by Bancerz et al. [13] in their study of lipases in the presence of bacterial (from *R. leguminosarum* bv. *trifolii* Rt24.2) and fungal (from *G. applanatum*) polysaccharides. The stabilising effect of polysaccharides has also been noted for other enzymes, such as laccase [11,32], cellobiose dehydrogenase [12], α-amylase [33], glucose oxidase [34], and horseradish peroxidase [35].

### 2.5. Effect of Temperature and pH on Laccase Activity and Stability in the Presence of EPS from S. borealis

**Figure 5:**
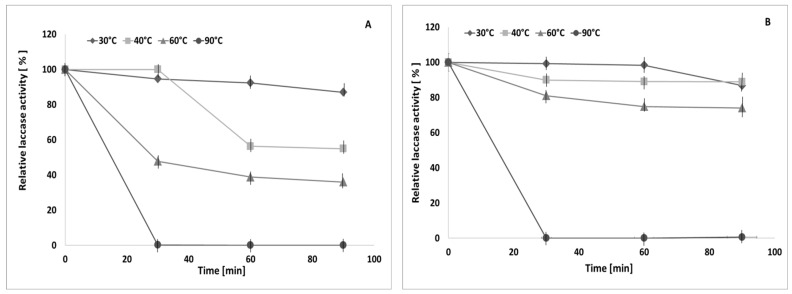
Effect of temperature on laccase stability without (control (**A**)) or with (**B**) EPS from *S. borealis.* The experiments were performed in triplicate. Average relative values (*n* = 3) and error bars are shown.

Enzymes have numerous biotechnological and industrial applications. However, one of the limitations of their use is the problem of maintaining their operational stability [36]. Due to their numerous bioactive properties, polysaccharides seem to be an interesting proposition as enzyme stabilising excipients [37,38]. In our previous studies, we demonstrated the possibility of using rhizobial exopolysaccharides as a natural stabiliser of *C. unicolor* laccase [10]. In turn, Chawla et al. [39] showed that laccase linked with chitosan had higher stability in a wider range of temperatures and pH compared to the non-immobilised enzyme. In the present paper, the influence of *S. borealis* exopolysaccharides on laccase activity and stability was determined at different temperature values. The incubation of the enzyme preparation and *S. borealis* EPS showed an influence of EPS on the stability of laccase compared to the control values (without EPS). In the samples incubated without exopolysaccharides, significantly reduced laccase activity (only 35.9% of basal activity retained at 60 °C and 54.9% of basal activity retained at 40 °C) was observed. The results shown in Figure 5B indicate that the presence of EPS from *S. borealis* in the probes preserves laccase activity, since the retained enzyme activity was 74.8% and 89% at 60 °C and 40 °C, respectively, after 90 min incubation.

**Figure 6:**
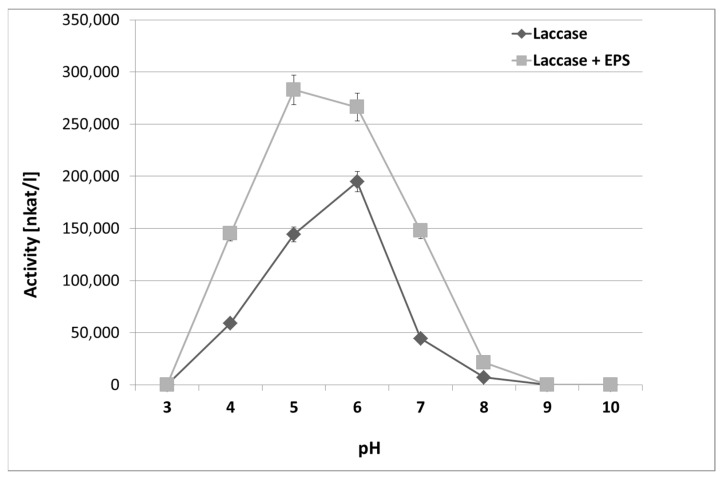
Effect of pH on laccase stability without (control) or with EPS from *S. borealis*. The experiments were performed in triplicate. Average relative values (*n* = 3) and error bars are shown.

The influence of the varying pH values (from 3.0 to 10.0) on laccase activity in the control samples without EPS and in the presence of *S. borealis* exopolysaccharides was also estimated (Figure 6). It was shown that the presence of EPS influenced laccase activity and shifted the pH optimum from 5.0 to 6.0, while the optimum pH for laccase is 5.0. Additionally, in the presence of EPS, the laccase activity was significantly higher than in samples without the polysaccharide; for example, at pH 5.0, the laccase activity with EPS was 300,000 nkat/L, while it was 150,000 nkat/L in the control without EPS.

### 2.6. Effect of S. borealis EPS on Laccase Stability in the Presence of Surfactants

**Figure 7:**
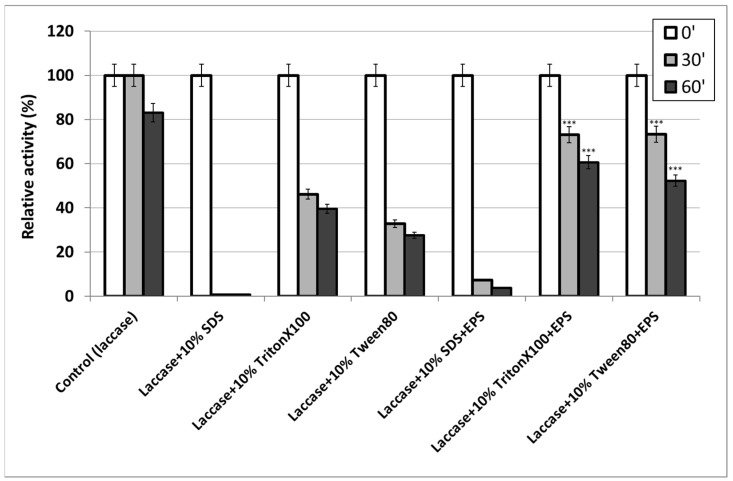
Laccase stability in the presence of surfactants and with or without EPS from *S. borealis*. The experiments were performed in triplicate. Average relative values (*n* = 3) and error bars are shown. Values with *p* ≤ 0.05 are significantly different (*** *p* ≤ 0.001).

Due to their good surface and structure-forming properties, protein–polysaccharide systems show higher efficiency and suitability for use in new industry technologies. Resistance to surfactants is very important in enzyme applications in the washing industry. It is considered that surfactants have a negative effect on enzymatic hydrolysis and are a competitive inhibitor in the reaction system [40]. The influence of surfactants on the activity of enzymes, e.g., lipase, has been studied to date [41]. In this paper, the effect of surfactants on laccase stability in the laccase–exopolysaccharide system was tested for the first time in the presence of anionic surfactant 10% SDS and two non-ionic surfactants, i.e., Tween-80 and Triton X-100. In the presence of the anionic surfactant (10% SDS), no significant laccase activity was observed, which was probably caused by the action of the strong surfactant on enzyme conformation and its irreversible effect on the protein structure. In turn, the analysis of laccase stability in the presence of Tween-80 and Triton X-100 showed lower laccase activity without EPS (32.8% relative laccase activity for Tween-80 and 46.2% relative laccase activity for Triton X-100) compared to preparations containing polysaccharides (73.3% relative laccase activity for Tween-80 and 73.1% relative laccase activity for Triton X-100) (Figure 7). This indicates the laccase-stabilising effect of the polysaccharide used in the presence of non-ionic surfactants.

## 3. Materials and Methods

### 3.1. Microorganisms and Culture Conditions

*Spongipellis borealis* (381) (KC293970) was deposited in the fungal collection at the Department of Biochemistry of Maria Curie-Skłodowska University (Lublin, Poland). The examined microorganism was stored on malt agar slants at 4 °C. The maternal cultures were cultivated for 14 days in a shaking system at 25 °C on liquid Oddoux medium composed of glucose 7 g/L, L-asparagine 0.5 g/L, MgSO_4_·7H_2_O, 0.5 g/L, KH_2_PO_4_ 0.5 g/L, malt extract 8 g/L, casein hydrolysate 1 g/L, thiamine 0.5 mg/L, riboflavin 0.5 mg/L, nicotinamide 0.5 mg/L, pyridoxine hydrochloride 0.5 mg/L, and calcium pantothenate 0.5 mg/L. The maternal mycelium homogenised with a blade homogeniser was used to inoculate the experimental cultures cultivated in 6 L flasks with 3.5 L of media. Then, 13-day submerged liquid fermentation was carried out at 26 °C using a Multitron incubator (Infors, Bottmingen, Switzerland) (agitation rate 120 rpm).

### 3.2. Genomic DNA Isolation and PCR Amplification of the ITS Region

The extraction procedure was performed according to the method described by Borges et al. [16] with minor modifications. *S. borealis* was grown on Lindeberg and Holm medium [15] at 25 °C for 10 days. The mycelium was washed twice with Tris EDTA buffer pH 7.5 and frozen in liquid nitrogen. PCRs were performed using Sigma RedTaq in a Tpersonal thermal cycler (Biometra, Goettingen, Germany). To confirm the identity of the fungus, the ITS region in the nuclear ribosomal repeat unit was determined by direct sequencing of the PCR products amplified with ITS1-ITS4 primers as described by White et al. and Gardes and Bruns [17,18].

### 3.3. Immunofluorescent Labelling of Cell Wall (1→3)-𝛼-D-Glucan

Fluorescently labelled antibodies were used to localise of (1→3)-𝛼-D-glucan in the cell wall of *S. borealis* according to the procedure described by Fujikawa et al. (2009) [42]. Fresh mycelium of *S. borealis* placed on Lab-Tek II Chamber slides (Nunc, Rochester, NY, USA) was fixed with a 3% (*v*/*v*) formaldehyde solution in distilled water at 65 °C for 30 min. The fixed fungal cells were washed in PBS buffer pH 7.4 before infiltration by 1% (*v*/*v*) Tween 20 in PBS buffer (PBS-T). To detect the presence of α-(1→3)-D-glucan, 150 µL of a solution of mouse IgM MOPC-104E (0.1 mg/mL in PBS buffer) (Sigma, St Louis, MO, USA) and 150 µL of Alexa Fluor 488 goat anti-mouse IgM (µ-chain specific) (0.1 mg/mL in PBS buffer) (Sigma, St. Louis, MO, USA) were used as primary and secondary antibodies, respectively. Observation was carried out using a fluorescence microscope, Olympus BX 51 (Olympus, Tokyo, Japan) at an excitation wavelength of 470/500 nm and an emission wavelength of 525/550 nm.

### 3.4. Extraction of Polysaccharides (EPS)

The *S. borealis* exopolysaccharides (EPS) were extracted from the fermentation broth using four volumes of cold 96% ethanol and stored overnight at 4 °C. The precipitated exopolysaccharides were collected by centrifugation at 9000 rpm for 10 min, dried at 60 °C to remove residual ethanol, and weighted. Next, the polysaccharide samples were dissolved in distilled water (1 mg/mL) and used for further testing. One part of the precipitated polysaccharides was deproteinated with 4% TCA. The sample was then centrifuged, and the liquid phase was separated. The polysaccharide was precipitated by adding 96% ethanol (1:1 ratio), and the precipitate of deproteinated polysaccharide was rinsed with acetone and centrifuged. The polysaccharide precipitate was dried.

### 3.5. Carbohydrate Analysis

Carbohydrate analysis was performed according to the procedure described in our previous publication [24]. The samples were hydrolysed to liberate monosaccharides using 2 M trifluoroacetic acid (100 °C, 5 h). Monosugars were converted into alditol acetates by reducing NaBD_4_ and peracetylation, as described elsewhere [43]. The position of sugar linkages in the polymer was established by methylation using methyl iodide, according to the method described by Ciukanu and Kerek (1984) [44]. Permethylated products were extracted into chloroform, dried under a nitrogen stream, and acid hydrolysed (2 M TFA, 100 °C, 5 h). The liberated, partly methylated products were N-acetylated, reduced with NaBD4, and peracetylated. The sugar derivatives were analysed by gas chromatography–mass spectrometry (GC–MS) carried out on an Agilent Technologies gas chromatograph (GC 7890A) connected to a mass selective detector (5975C inert XL EI/CI MSD) with a capillary column HP-5MS (30 m × 0.25 mm). 

FT-IR spectroscopy analysis was carried out with a Perkin Elmer FT-IR spectrophotometer (Model 1725X, Wellesley, MA, USA) in the wavelength range between 400 and 4000 cm^−1^.

### 3.6. Biochemical Determination of S. borealis Polysaccharides

The concentration of total carbohydrate in the fungal samples was analysed using the methodology based on phenol–sulphuric acid with D-glucose as a standard [45]. The protein concentration in the samples was determined using Bradford reagent [46]. The amount of phenolic compounds was determined with the DASA test using diazosulfanilamide (SA) as a reaction substrate [47].

### 3.7. Effect of S. borealis EPS on Proteolytic Enzyme Activity

The effect of EPS on proteolytic enzyme activity was determined by measuring the activity of pepsin, trypsin, and pycnoporopepsin enzymes in the presence of polysaccharides. The samples were prepared by mixing enzyme solutions with equal volumes of polysaccharides in a ratio of 1:1 (at the final concentration of 0.05%). The samples were incubated at 4 °C for 7 and 21 days. Proteolytic activity was assayed using the standard procedure with haemoglobin as a reaction substrate [48].

### 3.8. Laccase Activity Assay

Laccase activity was determined spectrophotometrically in a TECAN Infinite Pro200 plate-reader (TECAN, Maennedorf, Switzerland) with syringaldazine (Sigma-Aldrich, St. Louis, MO, USA) as a reaction substrate [49]. One nano katal (nkat) of laccase activity was defined as the amount of the enzyme catalysing the production of one nano mol of the coloured product (quinone, εM = 65,000 M^−1^ cm^−1^) per second at 25 °C and pH 5.5 and expressed as nano katals per litre of culture (nkat/L).

### 3.9. Effect of Temperature and pH on Laccase Activity and Stability in the Presence of EPS from S. borealis

The effect of exopolysaccharides (EPS) on laccase activity was determined by measuring enzyme activity in the presence of EPS isolated from *S. borealis*. The thermostability of laccase in the presence of EPS was determined by measuring the enzyme activity at various temperatures (30–90 °C) at pH 5.5. The effect of pH on laccase activity was assayed at 25 °C in a pH range of 3.0–10.0 using Robinson–Britton buffer. Laccase samples were prepared in microtubes by mixing the enzyme solutions with equal volumes of EPS in a ratio of 1:1 (at the final concentration of 0.05%). Samples containing distilled water instead of EPS were the controls. Laccase activity was determined using the standard procedure described above with syringaldazine as a reaction substrate.

### 3.10. Effect of S. borealis EPS on Laccase Stability in the Presence of Surfactants

To investigate the effect of the EPS on laccase stability in the presence of surfactants, aliquots of the enzyme in distilled water were incubated with equal volumes of EPS (at a final concentration of 0.05%). Then, the appropriate surfactant (10% Triton X-100, 10% Tween 80, or 10% SDS) was added to the laccase–EPS reaction mixture in a ratio of 1:1. The samples were incubated at 25 °C for 30 and 60 min. Controls were prepared as described previously, but the samples contained distilled water instead of EPS. Laccase activity was measured in standard assay conditions with syringaldazine as a reaction substrate.

### 3.11. Statistical Analysis

Data were analysed by one-way ANOVA test followed by Tukey’s post hoc test using Statistica 13 software (StatSoft Inc., Tulsa, OK, USA). All the results were expressed as mean ± SD from three experiments (*n* = 3). Values of *p* ≤ 0.05 were only reported as statistically significant.

## 4. Conclusions

In this study, the new exopolysaccharide (EPS) preparation isolated from laboratory-cultured vegetative mycelium of a new *Spongipellis borealis* strain isolated from the environment were characterised. Immunofluorescent labelling with specific antibodies was performed to detect (1→3)-α-D-glucan in *S. borealis* mycelium cell walls. Structural studies showed that the isolated biopolymer was composed mainly of glucose, galactose, and mannose monomers. The FT-IR analysis of the examined exopolysaccharide showed the presence of small amounts of protein and aromatic compounds, probably polyphenols, which was also confirmed by spectrophotometric methods. The GC–MS analysis of partly methylated alditol acetates revealed that the EPS contained mainly terminal, →3)-linked, →4)-linked, and →2,4)-linked hexoses. 

In the present study, the effect of *S. borealis* exopolysaccharides on the activity of proteolytic enzymes (pepsin, trypsin, and pycnoporopepsin) and laccase was determined for the first time. For proteolytic enzymes, an increase in their activity was detected after 21 days of incubation with EPS in comparison to the control without exopolysaccharide. The analysis of laccase stability in the presence of Tween-80 and Triton X-100 showed lower laccase activity without EPS compared to preparations containing polysaccharides. This indicates the laccase-stabilising effect of the polysaccharide used in the presence of non-ionic surfactants. Taking into account the improvement of the activity of the tested enzymes, and especially the storage stability in the presence of EPS, this experimental system can be proposed as a promising tool in biotechnological applications.

Summing up the results obtained in the present work, it can be suggested that the new EPS produced by the *S. borealis* strain isolated from the natural environment, subjected to qualitative analysis, seems to be a good candidate for a number of biotechnological processes. The possibility of using a polysaccharide preparation as a factor modifying the conditions of reactions carried out by selected biocatalysts (e.g., proteolytic enzymes or laccase) exhibiting different mechanisms of action seems to validate this thesis. From the practical application point of view, the easy standardisation of the production conditions, simple isolation procedure, and high efficiency of the described preparation is also very important in the perspective of increasing the scale of the process.

## Figures and Tables

**Table 1:** Percentage of sugar components in Sample 1 and 2 (after the proteinase treatment). The main components are bolded.

RT [min]	Sugar Component	Sample 1 [%]	Sample 2 [%]
14.067	ribose (Rib)	0.2	0.2
14.154	fucose (Fuc)	0.8	1.1
14.279	arabinose (Ara)	0.2	0.9
14.653	xylose (Xyl)	0.7	2.6
18.798	mannose (Man)	2.4	7.2
19.04	**glucose (Glc)**	**78.6**	**63.4**
19.147	**galactose (Gal)**	**16.4**	**24.6**
21.861	glucosamine (GlcN)	0.7	-

**Table 2:** Linkage analysis of sample 1 and sample 2 (after the proteinase treatment). The samples were methylated, hydrolysed, reduced, and peracetylated. The obtained permethylated alditol acetates were identified by GC–MS based on their mass spectra and retention times. The main components are marked in bold.

RT [min]	Type of Linkage	Sample 1 [%]	Sample 2 [%]
12.059	Hex-(1→	**16.8**	**12.9**
14.062	→3)-Hex I-(1→	**16.1**	**35.2**
14.130	→4)-Hex I-(1→	1.4	5.6
14.306	→4)-Hex II-(1→	**24**	5.1
14.491	→3)-Hex II-(1→	8.1	-
14.691	→6)-Hex I-(1→	4.6	-
15.354	→6)-Hex II-(1→	6.4	6
15.495	→2,6)-Hex-(1→	-	0.9
15.852	→4,6)-Hex-(1→	-	0.8
16.514	→2,3)-Hex-(1→	2.3	-
16.627	**→2,4)-Hex-(1→**	**16**	**30.4**
17.138	→2,4,6)-Hex-(1→	4.3	3.1

**Table 3:** Chemical composition of polysaccharides (EPS) obtained from *S. borealis*.

Samples	Protein [µg/mL]	Total Carbohydrates [µg/mL]	Reducing Carbohydrates [µg/mL]	Total Phenolic Compounds [µg/mL]
1	132.69 ± 8.6	673.85 ± 16.7	66.13 ± 3.4	26.14 ± 2.8
2	25.21 ± 3.4	363.88 ± 12.3	5.69 ± 1.1	9.89 ± 1.76

## Data Availability

Data is contained within the article.
